# Re-expression of miR-200s in claudin‐low mammary tumor cells alters cell shape and reduces proliferation and invasion potentially through modulating other miRNAs and SUZ12 regulated genes

**DOI:** 10.1186/s12935-021-01784-4

**Published:** 2021-02-04

**Authors:** K. Simpson, G. Conquer-van Heumen, K. L. Watson, M. Roth, C. J. Martin, R. A. Moorehead

**Affiliations:** Department of Biomedical Sciences, Ontario Veterinary College, University of Guelph, Guelph, ON Canada

**Keywords:** miR-200, Claudin‐low breast cancer, SUZ12, Migration, RNA sequencing, miRNA sequencing, H3K27me3

## Abstract

**Background:**

MicroRNAs are a class of non-coding RNAs that regulate gene expression through binding to mRNAs and preventing their translation. One family of microRNAs known as the miR-200 family is an important regulator of epithelial identity. The miR-200 family consists of five members expressed in two distinct clusters; the miR-200c/141 cluster and the miR-200b/200a/429 cluster. We have found that murine and human mammary tumor cells with claudin-low characteristics are associated with very low levels of all five miR-200s.

**Methods:**

To determine the impact of miR-200s on claudin-low mammary tumor cells, the miR-200c/141 cluster and the miR-200b/200a/429 cluster were stably re-expressed in murine (RJ423) and human (MDA-MB-231) claudin-low mammary tumor cells. Cell proliferation and migration were assessed using BrdU incorporation and transwell migration across Matrigel coated inserts, respectively. miRNA sequencing and RNA sequencing were performed to explore miRNAs and mRNAs regulated by miR-200 re-expression while Enrichr-based pathway analysis was utilized to identify cellular functions modified by miR-200s.

**Results:**

Re-expression of the miR-200s in murine and human claudin-low mammary tumor cells partially restored an epithelial cell morphology and significantly inhibited proliferation and cell invasion in vitro. miRNA sequencing and mRNA sequencing revealed that re-expression of miR-200s altered the expression of other microRNAs and genes regulated by SUZ12 providing insight into the complexity of miR-200 function. SUZ12 is a member of the polycomb repressor complex 2 that suppresses gene expression through methylating histone H3 at lysine 27. Flow cytometry confirmed that re-expression of miR-200s increased histone H3 methylation at lysine 27.

**Conclusions:**

Re-expression of miR-200s in claudin-low mammary tumor cells alters cell morphology and reduces proliferation and invasion, an effect potentially mediated by SUZ12-regulated genes and other microRNAs.

## Background

MicroRNAs (miRNAs) are small, non-coding RNA molecules that can be found in both introns and exons [1]. MiRNAs regulate mRNA translation by binding to mRNAs and in turn, target the mRNA for degradation or impair ribosome mediated translation of the mRNA [2, 3]. The interaction between miRNA and mRNA is primarily regulated by nucleotides 1–8 of the miRNA. This region, known as the seed sequence, binds to complementary sequences in the 3’ untranslated regions of mRNAs [4–12]. However, more recent studies have shown that non-canonical sites can also influence miRNA-mRNA interaction [13, 14] and miRNAs have been found to directly regulate gene transcription, activating Toll-like receptors and upregulating protein expression [15, 16], further increasing their complexity.

A number of miRNAs have been implicated in regulating breast cancer including the miR-200 family. The miR-200 family consists of 5 members organized into two clusters, the miR-200c/141 cluster and the miR-200b/200a/429 cluster [17–19]. miR-200b, miR-200c, and miR-429 share a common seed sequence (AA**U**ACUG) while miR-200a and miR-141 share the same seed sequence (AA**C**ACUG) that differs from the seed sequence of miR-200b, miR-200c and miR-429 by one nucleotide [20]. Therefore, both miR-200 clusters express miRNAs with the AA**U**ACUG and AA**C**ACUG seed sequences and thus are predicted to share mRNA targets. The most extensively characterized function of the miR-200 family is the regulation of epithelial and mesenchymal phenotypes. miR-200 family members negatively regulate mesenchymal transcription factors such as *Zeb1/2*, *Twist1/2*, *Snai1/2*, [19, 21–23] while inducing the expression of epithelial genes [24, 25]. Therefore, loss of miR-200s facilitates epithelial to mesenchymal transition (EMT).

Consistent with their roles in epithelial identity, most studies found that miR-200s are expressed in human luminal breast cancer but lost in triple negative breast cancer (TNBC) [26–30]. TNBCs lack expression of the estrogen receptor (ER), progesterone receptor (PR), and HER2, and have a poorer prognosis than luminal breast cancer [31–34]. Claudin-low breast cancers share features with TNBC including frequent loss of ER, PR and HER2 expression, high expression of mesenchymal genes and features of an epithelial–mesenchymal transition (EMT) phenotype [35]. In addition, claudin-low tumors express low levels of cell-cell adhesion and tight junction genes including claudins 3, 4, 7.

Our previous work with miR-200s in a murine claudin-low mammary tumor cell line, RJ423, revealed that re-expression of the miR-200b/200a/429 cluster reduced mesenchymal gene expression, restored a more epithelial-like morphology, reduced migration and inhibited tumor growth and metastasis in vivo [36]. In this current study, both the miR-200b/200a/429 and miR-200c/141 clusters were re-expressed in RJ423 cells as well as the human claudin-low breast cancer cell line, MDA-MB-231. Our data showed that when miR-200s are expressed at sufficiently high levels, they can restore a more epithelial like morphology and impair cell migration. miRNA sequencing revealed that re-expression of miR-200s significantly influenced the expression of > 50 miRNAs and RNA sequencing revealed significant alterations in > 1000 mRNAs. One of the molecular features associated with the differentially expressed mRNAs was an alteration in SUZ12 regulated genes. SUZ12 is a core protein of the polycomb repressor complex 2 PRC2 that regulates gene transcription through methylating histone H3 at lysine 27 (H3K27). Therefore, miR-200s appear to influence cell morphology and migration through (i) directly targeting mRNAs, (ii) altering the expression of other miRNAs, and (iii) regulating methylation of histone H3.

## Methods

### Cell lines and transfections

MCF-7 (cat #HTB-22) and MDA-MB-231 (cat #HTB-26) cells were purchased from ATCC (Manassas, VA). MDA-200c141 and MDA-200ba429 cells were created by transfecting MDA-MB-231 cells with pLenti 4.1 Ex miR200c-141 (cat #35,534, Addgene, Watertown, MA) or pLenti 4.1 Ex miR200b-200a-429 (cat #35,533, Addgene, Watertown, MA), respectively. MDA-231EV cells were created by infecting MDA-MB-231 cells with copGFP control lentiviral particles (cat #sc-108,084, Santa Cruz Biotechnology Inc, Dallas, TX). MCF-7, MDA-MB-231 and variants of MDA-MB-231 cells were maintained in DMEM media supplemented with 10% FBS, 1 mM sodium pyruvate, 4 mM glutamine, 1% antibiotic/antimycotic and 60 µg/ml puromycin (InvivoGen, San Diego, CA). RJ345 and RJ423 cells were created from mammary tumors that developed in distinct MTB-IGFIR transgenic mice and have previously been described [36–38]. RJ423-200c/141 cells were created by transfecting RJ423 cells with pLenti 4.1 Ex miR200c-141 (cat #35,534, Addgene, Watertown, MA). RJ423-200c/141 cells were cultured in DMEM media containing 10% FBS, 1 mM sodium pyruvate, 10 mM 4-(2-hydroxyethyl)-1-piperazineethanesulfonic acid (HEPES), 4 mM glutamine, 2 mM hydrocortisone, 5 µg/ml estrogen, 5 µg/ml prolactin, 10 µg/ml EGF, 10 µg/ml insulin, and 1% antibiotic-antimycotic (all supplements purchased from ThermoFisher Scientific, Waltham, MA) as well as 300 µg/ml of puromycin (InvivoGen, San Diego, CA). RJ345, RJ423EV and RJ423-200ba429 cells have been described previously as have the culture conditions for these cells [36].

### Crystal violet staining and transwell invasion assays

Crystal violet staining and transwell invasion assays were performed as described in [36] using 25,000 human tumor cells and 30,000 murine tumor cells for the transwell assays.

### RNA extraction, taqman qRT-PCR for microRNA expression and qRT-PCR for gene expression

RNA extraction, Taqman qRT-PCR and qRT-PCR for gene expression were performed as described in [37]. All miR-200 Taqman probes were obtained from ThermoFisher Scientific (Waltham, MA). miR-200 expression for the murine mammary tumor cell lines was normalized to sno202 and sno234 while the miR-200 expression of the human breast cancer cell lines was normalized to RNU44 and RNU48. All gene primers were obtained from Bio-Rad Laboratories (Mississauga, ON); *Cdh1* (qMmuCID0005843), *Hprt* (qMmuCED0045738), *Snai1* (qMmuCID0024342), *Snai2* (qMmuCED0046072), *Twist1* (qMmuCED0004065), *Twist2* (qMmuCID0009652) *Vim* (qMmuCID0005527), *Zeb1* (qMmuCID0009095), and *Zeb2* (qMmuCID0014662). *Hprt* was used as the housekeeping gene.

### miRNA sequencing

miRNA sequencing libraries were generated using NEB Multiplex small RNA library Prep Set for Illumina and sequencing quality was determined using an Agilent 2100 Bioanalyzer. Libraries were sequenced using an Illumina NextSeq 500 instrument. The Q30 scores for all samples were above 93%. Reads were then 3′-adaptor trimmed and filtered ≤ 15 bp reads with cutadapt software (v1.14). Trimmed reads were aligned to the reference genome with bowtie software. miRNA expression levels were calculated using mirdeep2 (v0.0.8) and differentially expressed miRNAs were performed with edgeR (v3.18.1). Library preparation, sequencing and data analysis were performed by Arraystar Inc. (Rockville, MD). Four independent samples were sequenced.

### RNA sequencing

RNA sequencing for one set of RJ423EV samples and the RJ423ba429 samples was performed at the Genome Quebec Innovation Centre at McGill University using the Illumina Hiseq 2500 v4 PE125 as previously described [37]. RNA sequencing for a second set of RJ423EV samples as well as RJ423-200c/141, MDAEV, MDA-200c/141 and MDA-200ba429 were performed by Arraystar Inc (Arraystar Inc., Rockville MD). All Fastq files were processed using Genialis software (Genialis Inc, Houston, TX) following the standard RNA-seq pipeline which uses BBDuk to remove adapters and trim reads, STAR to align the reads, and feature counts to generate gene level counts. RNA sequencing has been uploaded to GEO under accession number GSE150107. Note that our original data for RJ423EV and RJ423-200ba429 samples found at GSE113162 [36] were analyzed by the Genome Quebec Innovation Centre at McGill University and thus might differ from the data in this manuscript that was analyzed using Genialis software. Three independent samples were sequenced for the RJ423 variants and four independent samples were sequenced for the MDA-231 variants.

### BrdU and H3K27me3 flow cytometry

For the murine cell lines, a FITC BrdU flow kit (BD Biosciences, San Jose, CA, cat #559,619) and for the human cells an APC BrdU flow kit (BD Biosciences, San Jose, CA, cat #552,598) were used following the manufacturer’s protocol. The APC kit was required for the human cell lines as MDA-231EV cell lines express GFP. Briefly, cells were incubated with 1 mM BrdU in fully supplemented media for 45 min. Cells were then fixed, washed and analyzed on an Accuri C6 cytometer (BD Biosciences, San Jose, CA) using a flow rate of 35 μl/min and 20,000 events were collected. For the H3K27me3 analysis, cells were fixed in 4% paraformaldehyde for 15min and then permeabilized in 90% methanol for 10 min. Cells were then incubated with a 1:200 dilution of anti-H3K27me3 (Cell Signaling cat #9733S) or IgG KP isotype control (Cell Signaling cat #3900s) for 1 h followed by a 30 min incubation with the appropriate secondary antibody (Alexa Fluor 488 for the mouse cells and APC for the human cells). Cells were then resuspended in 5% 7-AAD solution for 5 min. Cells were analyzed on an Accuri C6 cytometer (BD Biosciences, San Jose, CA) using a flow rate of 35 μl/min and 20,000 events were collected.

### Venn diagrams

Venn diagrams were generated using Venny 2.1.0 [38].

### KEGG pathway, gene ontology and ChEA

For RNA sequencing data Enhrichr [39, 40] was used to identify KEGG and Gene Ontology Pathways affected as well as transcription factors implicated in regulating the differentially expressed genes (ENCODE and ChEA Consensus TFs from ChIP-X).

### miRNA binding site analysis

Mouse genes with a target score ≥ 50 for either the miR-200c/141 cluster (miR-200c-3p, miR-200c-5p, miR-141-3p, miR-141-5p) or the miR-200b/200a/429 cluster (miR-200a-3p, miR-200a-5p, miR-200b-3p, miR-200b-5p, miR-429-3p, miR-429-5p) were identified in miRDB [41, 42] and compared to the mRNAs differentially expressed in the RNA-seq data of RJ423c141 or RJ423ba429 cells. The same approach was used for the human genes.

### Statistics

Statistical significance was determined using an ANOVA followed by a Dunnett’s test using GraphPad Prism 9 (San Diego, CA). For the MCF-7 and MDA-231 variants, all comparisons were made using MDA-231EV cells as the control and for the RJ345 and RJ423 variants, all comparisons were made using RJ423EV cells as the control.

## Results

### miR-200 expression and the impact on mesenchymal gene expression and cell shape

**Fig. 1:**
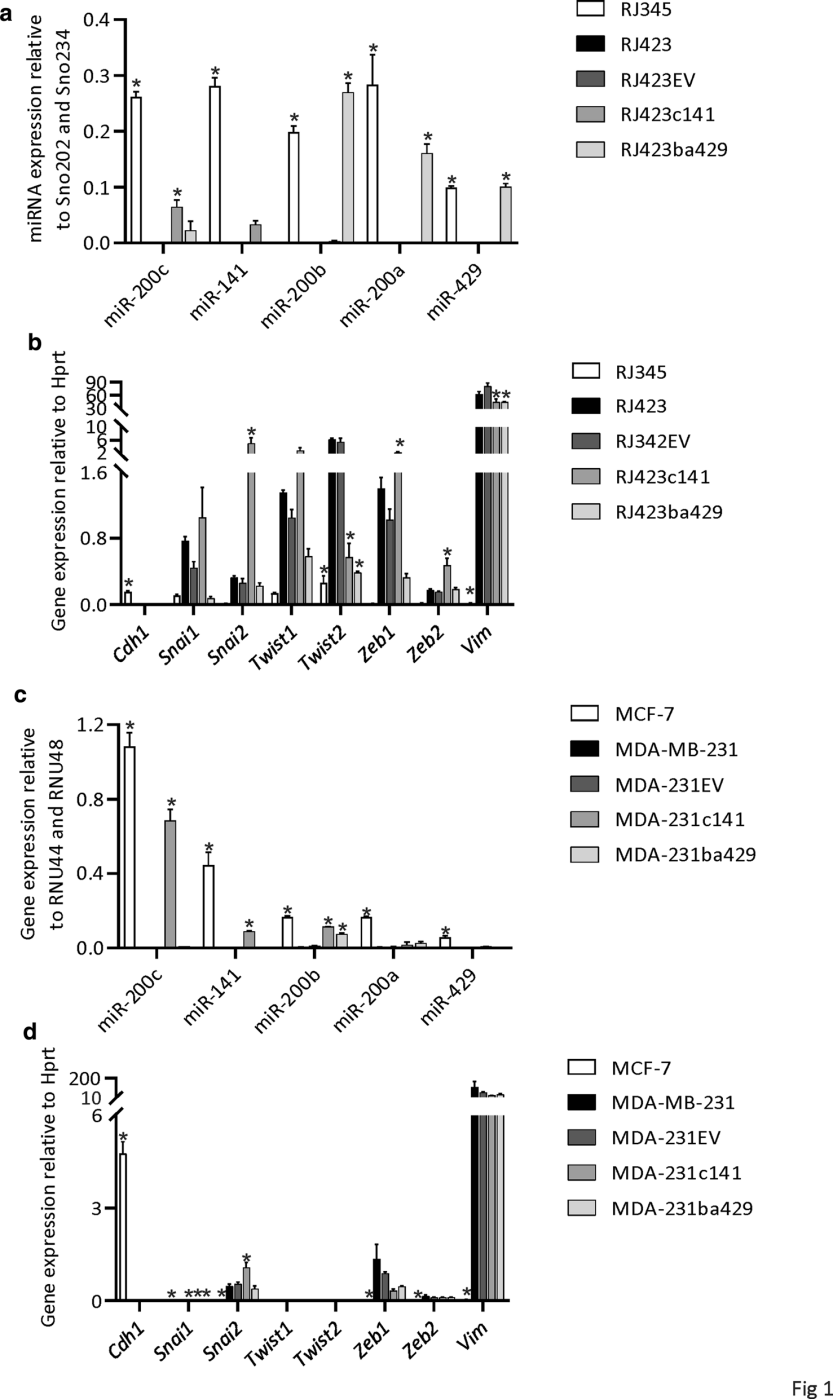
MicroRNA and gene expression. Expression of the miR-200 family (**a**, **c**) and epithelial and mesenchymal genes (**b**, **d**) in RJ345, RJ423, RJ423EV, RJ423c141 and RJ423ba429 cells (**a**, **b**) and MCF-7, MDA-MB-231, MDA-231EV, MDA-231c141 and MDA-231ba429 cells (**c**, **d**). miR-200 expression was determined using Taqman qRT-PCR while gene expression was determined using qRT-PCR. The graphs represent the mean ± SEM (n = 3) and the * indicates as significant difference (p < 0.05) from RJ423EV cells (**a**, **b**) or MDA-231EV cells (**c**, **d**)

As shown in Fig. 1, the murine (RJ345, Fig. 1a) and human (MCF-7, Fig. 1c) mammary tumor cell lines with epithelial characteristics expressed high levels of all 5 miR-200 family members compared to the murine (RJ423/RJ423EV) and human (MDA-MB-231/MDA-231EV) claudin-low mammary tumor cell lines, respectively. Transfection of either the miR-200c/141 cluster or miR-200b/200a/429 cluster in RJ423 or MDA-MB-231 cells significantly increased the expression of one or more of the appropriate miR-200s. For example, RJ423ba429 cells expressed significantly higher levels of miR-200b, miR-200a and miR-429 compared to RJ423EV and MDA-231c141 cells expressed significantly higher levels of miR-200c and miR-141 than MDA-231EV cells.

Since the miR-200 family has been implicated in regulating epithelial to mesenchymal transition (EMT) [19, 21–25], the expression of the epithelial gene *Cdh1*, and several mesenchymal genes were evaluated. The altered miR-200 expression in RJ423 and MDA-MB-231 cells did not significantly increase the expression of the epithelial gene, *Cdh1* and had inconsistent effects on mesenchymal genes (Fig. 1b, d). Only *Twist2* and *Vim* were significantly reduced by miR-200 expression in RJ423 cells (Fig. 1c) and only *Snai1* was significantly reduced by miR-200 expression in MDA-MB-231 cells (Fig. 1d) however, the expression of *Snai1* was extremely low in all the cell lines. Somewhat surprisingly, re-expression of miR-200c/141 in both RJ423 and MDA-MB-231 cells induced a significant increase in *Snai2* expression (Fig. 1b, d). *Zeb1*, *Zeb2* are predicted targets of all five miR-200s while *Snai2* is a predicted target of miR-200b, miR-200c and miR-429 according to miRDB (mirdb.org). *Zeb1* was reduced approximately twofold in both RJ423ba429 cells compared to RJ423EV cells and MDA-231c141 cells compared to MDA-231EV cells but these differences did not reach statistical significance. The mesenchymal genes evaluated in this study are regulated by other miRNAs in addition to the miR-200 family and several transcription factors and thus it is the balance of transcriptional induction and repression as well as post-transcriptional regulation that would determine gene expression levels.

**Fig. 2:**
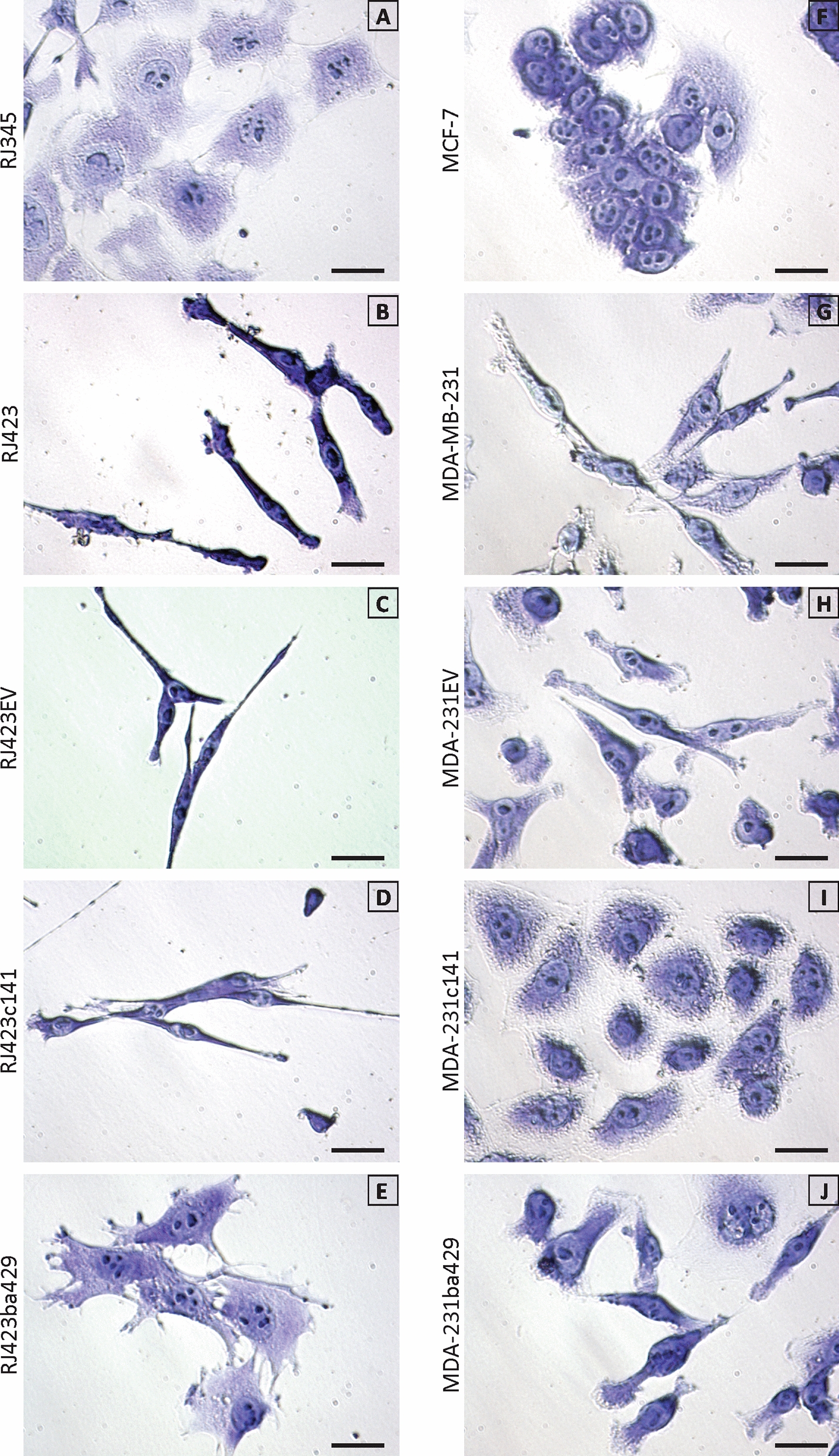
Cell morphology. Crystal violet staining of murine (**a**–**e**) and human (**f**–**j**) mammary tumor cells. Mammary tumor cells with a luminal phenotype (**a**, **f**) display a more rounded/cuboidal morphology while mammary tumor cells with a claudin-low phenotype (**b**, **g**) display a more spindle-like morphology. Re-expression of the miR-200b/200a/429 cluster in RJ423 cells (**e**) and the miR-200c/141 cluster in MDA-MB-231 cells (**i**) reverted these cells to a more luminal morphology. Scale bars are 25 µm

During routine culture of these cells it was observed that RJ345 (Fig. 2a) and MCF-7 (Fig. 2f) cells displayed a more rounded cell shape typically associated with epithelial cells while RJ423 (Fig. 2b) and MDA-MB-231 (Fig. 2g) cells have a more spindle shaped morphology typically associated with mesenchymal cells. Re-expression of miR-200b/200a/429 in RJ423 cells (Fig. 2e and [36]) and re-expression of miR-200c/141 in MDA-MB-231 cells (Fig. 2i) reverted these cells to a more epithelial morphology. This morphological change was observed in all RJ423ba429 (Fig. 2e) and MDA-231c141 (Fig. 2i) cells and was maintained when the cells were passaged.

**Fig. 3:**
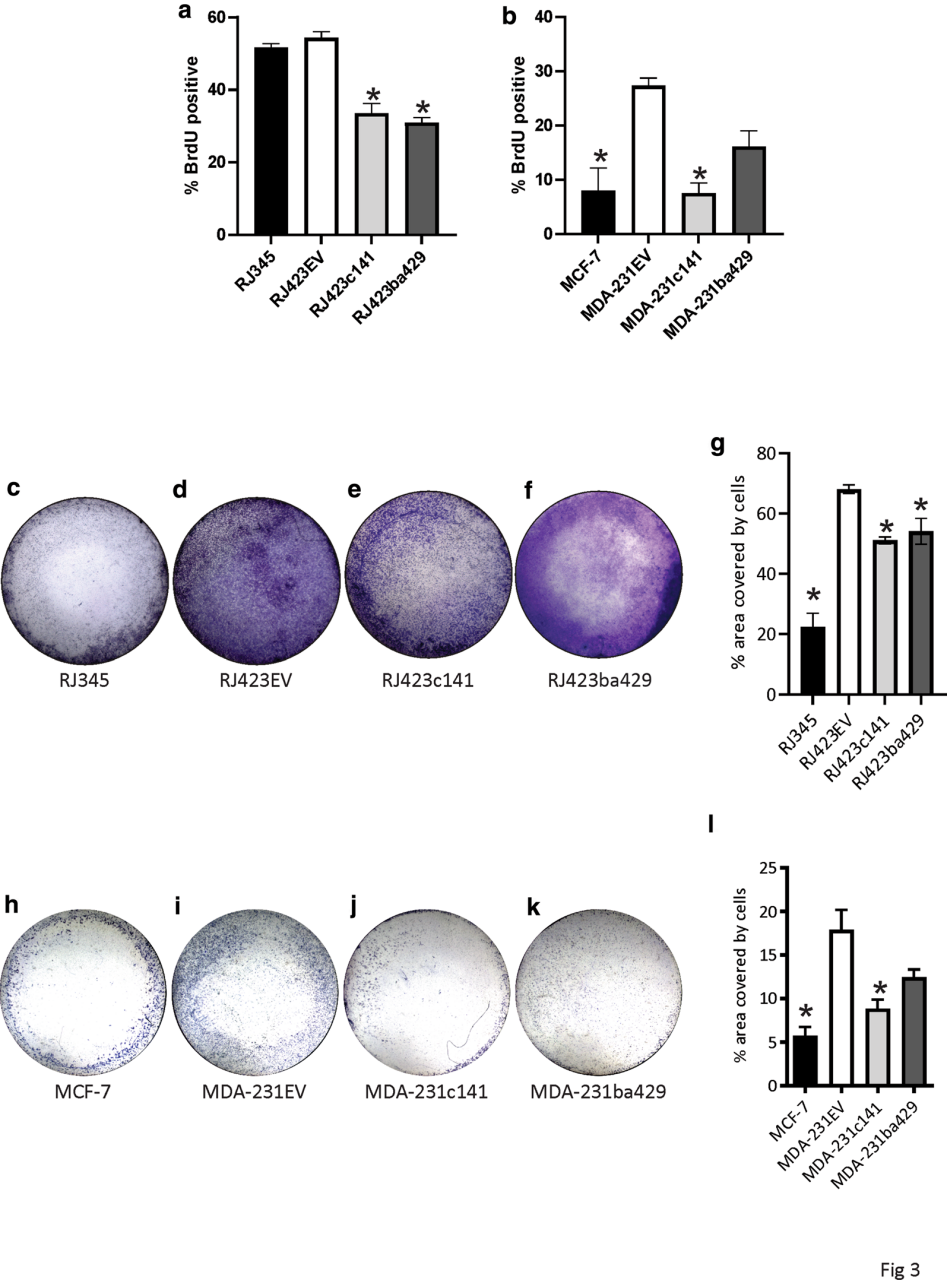
Cell proliferation and invasion. Proliferation (**a**, **b**) and transwell migration across Matrigel coated inserts (**c**–**l**) in murine (**a**, **c**–**g**) and human (**b**, **h**–**l**) mammary tumor cells. Proliferation was determined using BrdU incorporation while transwell migration was determined by calculating the percentage of the lower transwell membrane covered with crystal violet stained cells. Representative images of the crystal violet stained cells on the bottom of the transwell insert have been provided for murine (**c**–**f**) and human (**h**–**k**) mammary tumor cells. Graphs (**a**, **b**, **g**, **l**) represent mean ± SEM with n = 3 for the BrdU assay and n = 4 for the transwell assay. * Indicate values significantly different (p < 0.05) from either RJ423EV or MDA-231EV cells

To evaluate whether miR-200 re-expression altered cell proliferation, BrdU incorporation was assessed using flow cytometry. Re-expression of miR-200c/141 or miR-200b/200a/429 in murine tumor cells significantly reduced proliferation (Fig. 3a). In the MDA-MB-231 cells re-expression of the miR-200c/141 cluster but not the miR-200b/200a/429 cluster significantly reduced proliferation in MDA-MB-231 cells (Fig. 3b).

To determine the impact of miR-200s on migration, matrigel-coated, transwell assays were performed. In RJ423 cells, re-expression of either the miR-200c/141 cluster or the miR-200b/200a/429 cluster significantly reduced cell migration and invasion however, these cells still migrated and invaded more efficiently than the RJ345 cells (Fig. 3c–g). Re-expression of the miR-200c/141 cluster but not the miR-200b/200a/429 cluster in MDA-MB-231 cells significantly reduced migration and invasion and re-expression of the miR-200c/141 cluster returned migration and invasion capacity to a level similar to MCF-7 cells (Fig. 3h–l).

### Re-expression of miR-200s influence the expression of other miRNAs

miRNA sequencing was performed to confirm that the appropriate miR-200 family members had been upregulated in RJ423c141, RJ423ba429, MDA-231c141 and MDA-231ba429 cells and to determine whether re-expression of miR-200s influenced the expression of other miRNAs. The complete miRNA sequencing files for each cell line compared to their respective control line are presented as Additional file 1 and the top 10 upregulated and downregulated miRNAs based on p-values are presented in Table 1. In each cell line, the miR-200s expressed by the transfected plasmid were found within the top 10 upregulated miRNAs confirming appropriate miR-200 expression. In contrast to the qPCR data (Fig. 1a, c), miRNA-seq found a significant increase in miR-200a and miR-429 in the MDA-231ba429 cells compared to the MDA-231EV cells. The miRNA sequencing data also found a significant increase in miR-200c in RJ423ba429 cells and significant increase in miR-200b and miR-429 in MDA-231c141 cells (Additional file 1).

**Table 1:** Top 10 upregulated and downregulated miRNAs as determined by miRNA-seq

Upregulated miRNAs	log2FC	p-value	Downregulated miRNAs	log2FC	p-value
MDA-231c141 vs. MDA-231EV					
hsa-miR-200c-3p	7.1	0	hsa-miR-335-3p	− 12.2	0
hsa-miR-129-5p	3.8	3.1 × 10^−65^	hsa-miR-452-5p	− 9.5	0
hsa-miR-141-3p	5.3	1.5 × 10 ^−58^	hsa-miR-34c-5p	− 3.9	1.4 × 10^−136^
hsa-miR-139-5p	2.2	5.4 × 10^−50^	hsa-miR-34a-5p	− 3.8	1.1 × 10^−67^
hsa-miR-1301-3p	2.6	3.4 × 10^−47^	hsa-miR-210-3p	− 2.8	4.1 × 10^−63^
hsa-miR-30a-5p	1.6	6.0 × 10^−46^	hsa-miR-335-5p	− 7.9	1.1 × 10^−59^
hsa-miR-194-5p	2.2	1.3 × 10^−45^	hsa-miR-27b-5p	− 2.4	9.0 × 10^−58^
hsa-miR-192-5p	2.3	7.8 × 10^−43^	hsa-miR-23b-3p	− 1.7	2.6 × 10^−43^
hsa-miR-455-5p	1.8	1.3 × 10^−40^	hsa-miR-34b-3p	− 4.4	1.4 × 10^−41^
hsa-miR-7156-5p	5.2	2.1 × 10^−35^	hsa-miR-10399-3p	− 2.5	2.1 × 10^−39^
MDA-231ba429 vs. MD-231EV					
hsa-miR-200b-3p	2.9	3.7 × 10 ^−71^	hsa-miR-27b-3p	− 1.7	7.6 × 10^−59^
hsa-miR-200a-3p	3.2	4.1 × 10 ^−48^	hsa-miR-1269b	− 8.7	6.2 × 10^−40^
hsa-miR-200a-5p	3.2	2.3 × 10 ^−32^	hsa-miR-3681-5p	− 3.9	3.1 × 10^−39^
hsa-miR-200b-5p	2.9	1.1 × 10 ^−26^	hsa-miR-34a-5p	− 3.5	5.3 × 10^−39^
hsa-miR-30c-2-3p	1.1	2.0 × 10^−17^	hsa-miR-27b-5p	− 2.1	1.2 × 10^−34^
hsa-miR-708-5p	4.8	9.1 × 10^−17^	hsa-miR-125b-2-3p	− 6.1	4.6 × 10^−33^
hsa-miR-218-5p	1.2	4.2 × 10^−14^	hsa-miR-196a-5p	− 3.5	1.4 × 10^−19^
hsa-miR-449c-5p	1.8	1.6 × 10^−13^	hsa-miR-95-3p	− 2.5	4.5 × 10^−18^
hsa-miR-3074-5p	1.6	1.1 × 10^−11^	hsa-miR-516a-5p	− 4.0	1.3 × 10^−17^
hsa-mir-429	1.7	3.6 × 10 ^−11^	hsa-miR-23b-3p	− 1.4	9.9 × 10^−17^
RJ423c141 vs. RJ423EV					
mmu-miR-200c-3p	5.9	3.9 × 10 ^−51^	mmu-miR-365-5p	− 8.3	3.4 × 10^−13^
mmu-miR-615-3p	8.0	5.0 × 10^−43^	mmu-miR-132-3p	− 3.1	6.5 × 10^−12^
mmu-miR-146b-5p	2.5	4.9 × 10^−13^	mmu-miR-212-5p	− 4.0	7.3 × 10^−10^
mmu-miR-99a-5p	2.1	4.1 × 10^−12^	mmu-miR-1983	− 1.9	6.9 × 10^−9^
mmu-miR-378c	2.6	7.6 × 10^−12^	mmu-miR-6540-5p	− 8.0	1.2 × 10^−7^
mmu-miR-378d	2.4	7.6 × 10^−12^	mmu-miR-212-3p	− 4.7	4.2 × 10^−6^
mmu-miR-378a-3p	2.3	2.1 × 10^−11^	mmu-miR-210-3p	− 1.3	5.4 × 10^−5^
mmu-miR-125b-2-3p	1.9	7.1 × 10^−9^	mmu-miR-124-3p	− 3.0	2.3 × 10^−4^
mmu-miR-141-5p	5.9	2.5 × 10 ^−8^	mmu-miR-582-3p	− 2.5	2.7 × 10^−4^
mmu-miR-141-3p	4.8	7.4 × 10 ^−8^	mmu-miR-582-5p	− 3.1	2.8 × 10^−4^
RJ423ba429 vs. RJ423EV					
mmu-miR-204-5p	10.4	1.5 × 10^−73^	mmu-miR-346-5p	− 11.9	7.6 × 10^−59^
mmu-miR-196a-5p	7.5	1.7 × 10^−71^	mmu-miR-199b-5p	− 5.7	6.2 × 10^−40^
mmu-miR-200b-3p	9.2	1.2 × 10 ^−55^	mmu-miR-9-5p	− 4.4	3.1 × 10^−39^
mmu-miR-200a-5p	8.5	2.5 × 10 ^−54^	mmu-miR-330-5p	− 4.3	5.3 × 10^−39^
mmu-miR-200b-5p	8.6	9.4 × 10 ^−30^	mmu-miR-1983	− 2.9	1.2 × 10^−34^
mmu-miR-196a-1-3p	8.9	9.3 × 10^−28^	mmu-miR-199a3p	− 2.4	4.6 × 10^−33^
mmu-miR-211-5p	7.0	3.8 × 10^ 21^	mmu-miR-199b-3p	− 2.4	1.4 × 10^−19^
mmu-miR-200a-3p	8.9	2.1 × 10 ^−19^	mmu-miR-146a-5p	− 3.3	4.5 × 10^−18^
mmu-miR-204-3p	9.5	3.3 × 10^−19^	mmu-miR-138-5p	− 3.8	1.3 × 10^−17^
mmu-miR-429-3p	9.7	3.6 × 10 ^−18^	mmu-miR-9-3p	− 3.9	9.9 × 10^−17^

To further explore miRNAs that were unique and shared in RJ423ba429 cells compared to RJ423c141 cells and MDA-231c141 cells compared to MDA-231ba429 cells, Venn diagrams were created (Additional file 2). These diagrams show miRNAs that were significantly, differentially expressed (log2FC ≥ 1, p < 0.01) in RJ423c141 and RJ423ba429 cells compared to RJ423EV cells (Additional file 2A) and MDA-231c131 and MDA-231ba429 cells compared to MDA-231EV cells (Additional file 2B). Re-expression of the miR-200b/200a/429 cluster altered the expression of more miRNAs than re-expression of the miR-200c/141 cluster in RJ423 cells and only a small number of miRNAs were shared (Additional file 2A). In the human cancer cells, re-expression of the miR-200c/141 cluster altered more miRNAs than re-expression of the miR-200b/200a/429 cluster and like the murine cells, a relatively small proportion of miRNAs were shared (Additional file 2B). Additional file 2C illustrates the number shared and unique miRNAs in the mouse and human miR-200 modified cells.

### Re-expression of miR-200s altered the expression of genes regulated by SUZ12

**Fig. 4:**
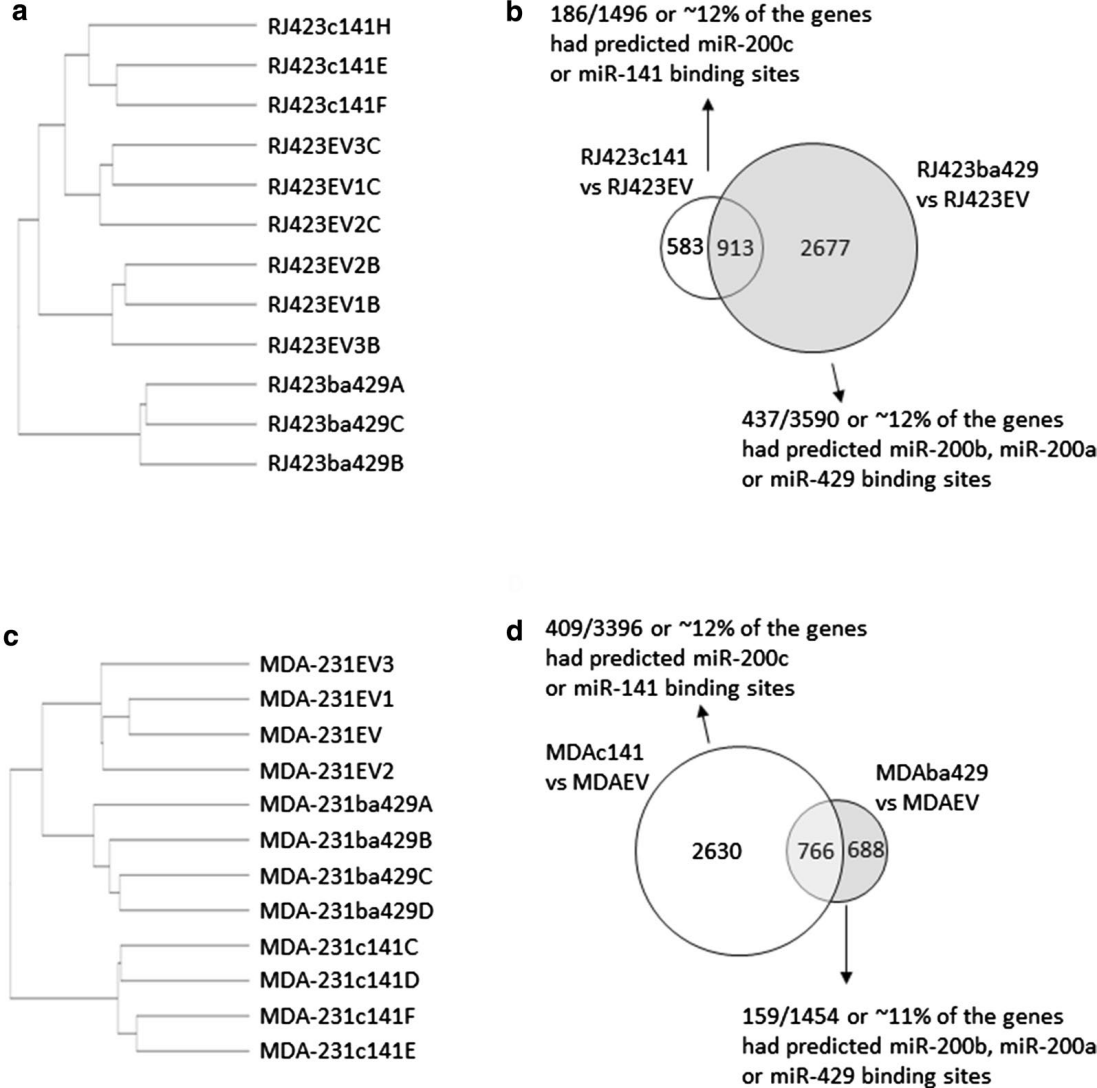
Hierarchical clustering of RNA sequencing data (n = 4). Hierarchical clustering (**a**, **c**) and Venn diagram (**b**, **d**) analysis of mRNAs differentially expressed in RJ423c141 and RJ423ba429 cells relative to RJ423EV cells (**a**, **b**) as well as in, MDA-231c141 and MDA-231ba429 cells relative to MDA-231EV cells (**c**, **d**). The Venn diagrams also indicate the percentage of differentially expressed genes with predicted miR-200 binding sites as determined by the miRDB database

RNA sequencing was also performed to identify genes altered by miR-200s. Hierarchical clustering revealed that the cell types segregated into discrete clusters and RJ423c141 cells clustered more closely with RJ423EV cells than RJ423ba429 cells (Fig. 4a). The analysis of this data was complicated by the fact that the RNA sequencing for the RJ423c141 and RJ423ba429 cells was performed on different days. In each RNA sequencing run, the same RNA from RJ423EV cells was used. However, the hierarchical clustering reveals some batch effects from the different runs as the two sets of RJ423EV samples (RJ423EVB samples that were utilized with the RJ423ba49 cells and RJ423EVC samples that were utilized with the RJ423c141 cells) showed distinct RNA expression profiles (Fig. 4b). Using a of log2FC ≥ 1 and FDR ≤ 0.01, 494 genes were upregulated and 1002 genes were downregulated in RJ423c141 cells compared to RJ423EV cells and 186 (~ 12%) of these genes had predicted miR-200c or miR-141 binding sites (using miRDB, Fig. 4b). When RJ423ba429 cells were compared to RJ423EV cells, 1691 genes were upregulated and 1899 were downregulated and 437 (~ 12%) of these genes had predicted miR-200b, 200a or miR-429 binding sites (Fig. 4b). Of the genes differentially regulated in RJ423c141 vs. RJ423EV and RJ423ba429 vs. RJ423EV, 913 were shared and 583 and 2677 were unique to RJ423c141 and RJ423ba429 cells, respectively (Fig. 4b).

RNA sequencing was also performed on MDA-231EV, MDA-231c141 and MDA-231ba429 cells. Again, hierarchical clustering revealed that the 3 cell types segregated into discrete clusters and MDA-231b429 cells were more similar to MDA-231EV cells than MDA-231c141 cells (Fig. 4c). Using a of log2FC ≥ 1 and FDR ≤ 0.01, 1733 genes were upregulated, and 1663 genes were downregulated in MDA-231c141 cells compared to MDA-231EV cells and 409 (~ 12%) have predicted miR-200c or miR-141 binding sites (Fig. 3d). When MDA-231ba429 cells were compared to MDA-231EV cells, 506 genes were upregulated and 948 were downregulated and 159 (~ 11%) of these genes had predicted miR-200b, miR-200a or miR-429 binding sites (Fig. 4d). Of the genes differentially regulated in MDA-231c141 vs. MDA-231EV and MDA-231ba429 vs. MDA-231EV, 766 were shared and 2630 and 688 were unique to MDA-231c141 and MDA-231ba429 cells, respectively (Fig. 4d).

**Fig. 5:**
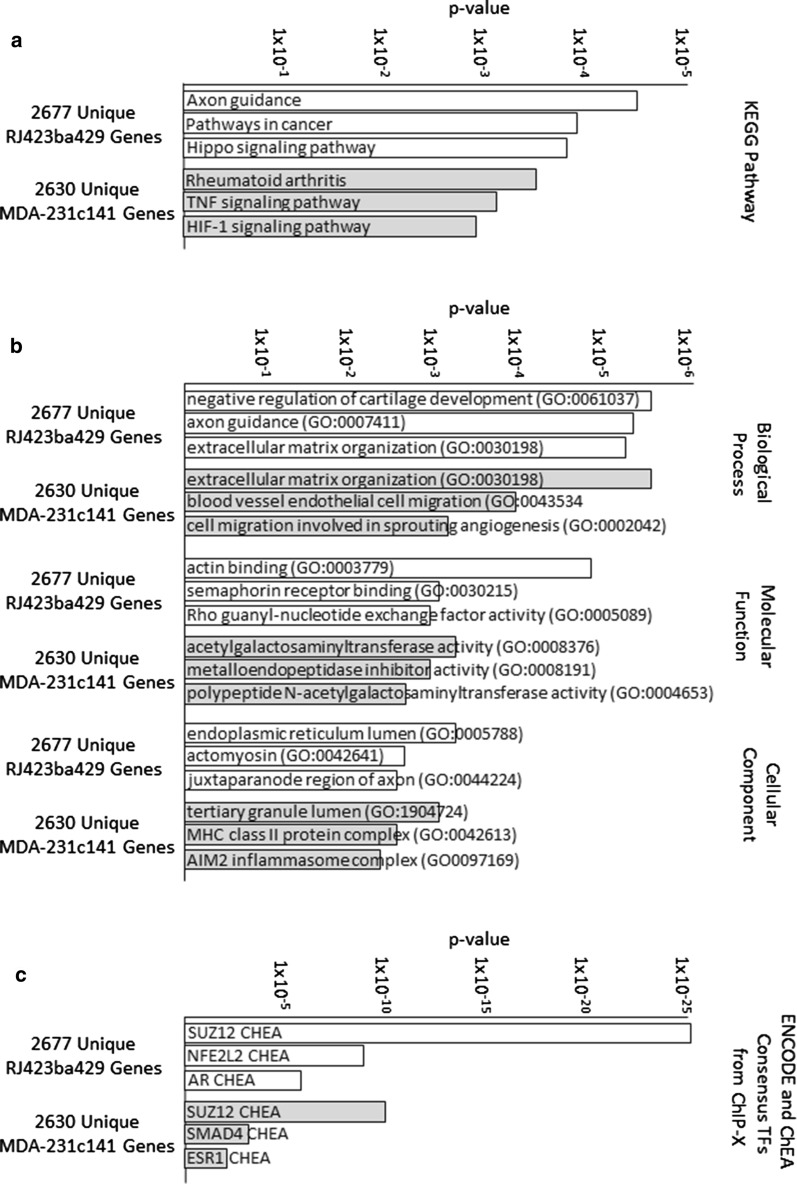
Pathway analysis of genes regulated by miR-200s. KEGG Pathways (**a**), Gene Ontology (**b**), and ENCODE/ChEA analysis (**c**) of genes differentially expressed in RJ423ba429 cells (white bars) or MDA-231c141 cells (grey bars)

Since RJ423ba429 cells and MDA-231c141 cells display a change in cell morphology (Fig. 2), the genes unique to these two cell lines were further examined using Enrichr [40, 41] to identify pathways associated with the unique genes in RJ423ba429 and MDA-231c141 cells (Fig. 5a, b). Although there were only a small number of pathways shared between RJ423ba429 and MDA-231c141 cells, the top ChEA category was the same in both cell lines, SUZ12 (Fig. 5c). The Encode and ChEA Consensus TFs from ChIP-X database from Enrichr lists 4439 predicted Suz12 targets and it was found that 863 of the 2677 (~ 32%) unique genes in RJ423ba429 cells and 706 of the 2630 (~ 27%) unique genes in MDA-231c141 cells were predicted targets of SUZ12.

**Fig. 6:**
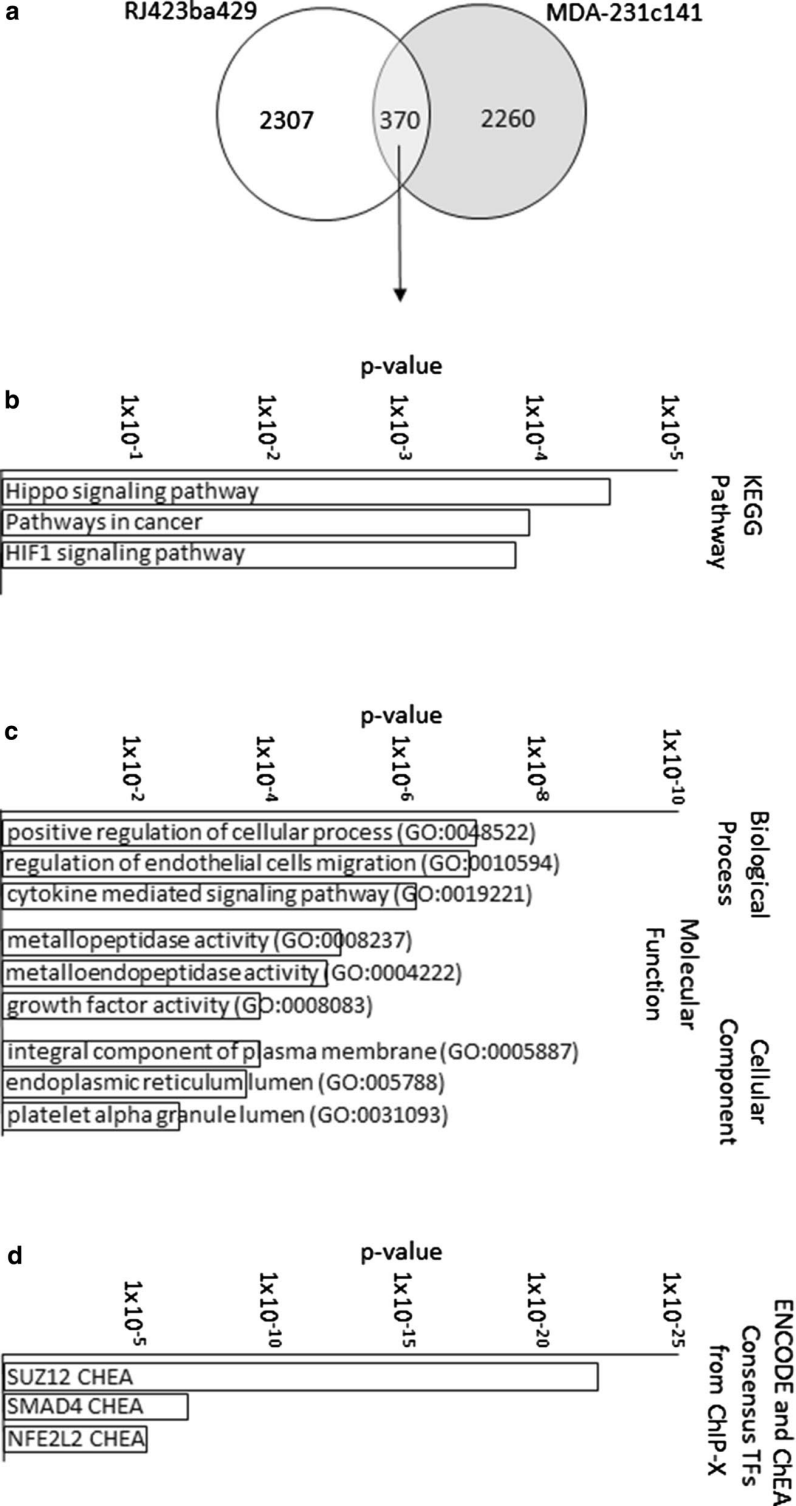
Venn diagrams and pathway analysis of shared genes. Venn diagram (**a**), KEGG Pathways (**b**), Gene Ontology (**c**) and ENCODE/ChEA analysis (**d**) of genes differentially expressed in both RJ423ba429 and MDA-231c141 cells

When comparing the RJ423ba429 unique gene profile to the MDA-231c141 unique gene profile, it was determined that 370 genes were shared between the unique gene profiles of the two cell lines (Fig. 6a). The KEGG and Gene Ontology Pathways as well as the ChEA pathways of these shared genes are presented in Fig. 5b, c and suggest that altering miR-200 expression in these cells influences pathways such as hippo signaling, endothelial migration and metallopeptidase activity (Fig. 6d). The top ChEA pathways remained SUZ12 and 194 of the 370 shared genes (~ 52%) were predicted SUZ12 targets.

### H3K27me3 levels are elevated in RJ423ba429 and MDA-231c141 cells

**Fig. 7:**
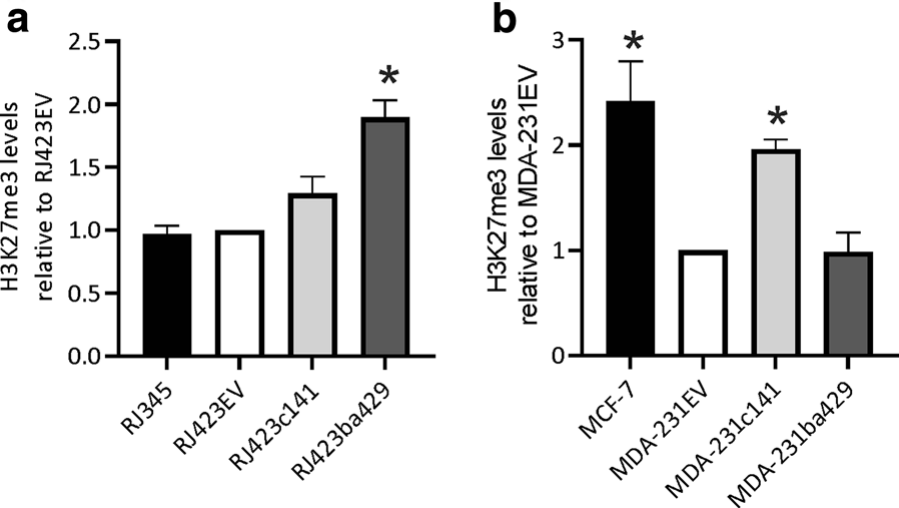
Quantification of H3K27me3 levels using flow cytometry. The levels of H3K27me3 in RJ345, RJ423c141, and RJ423ba429 cells were expressed relative to the levels of H3K27me3 in RJ423EV cells (**a**) while the levels of H3K27me3 in MCF-7, MDA-231c141 and MDA-231ba429 cells were expressed relative to the levels of H3K27me3 in MDA-231EV cells (**b**). The graphs represent the mean ± SEM (n = 5) and the * indicates p < 0.05 relative to RJ423EV cells (**a**) and MDA-231EV cells (**b**)

SUZ12 is a member of the polycomb repressor complex 2 (PRC2) which mono-, di- and tri-methylates lysine 27 on histone H3 [43–50]. To further investigate differences in SUZ12 activity, flow cytometry was used to quantify trimethylated histone H3 (H3K27me3). As shown in Fig. 7, RJ423ba429 cells had significantly higher levels of H3K27me3 than RJ423EV cells (Fig. 7a) and MDA-231c141 cells had significantly higher levels of H3K27me3 than MDA-231EV cells (Fig. 7b). The luminal breast cancer cell line, MCF-7, also had significantly elevated levels of H3K27me3 compared to the claudin-low breast cancer cell line MDA-231EV while RJ345 cells that have both luminal and basal-like characteristics, did not have higher levels of H3K27me3 compared to the claudin-low RJ423EV cells.

## Discussion

Re-expression of miR-200s in human and murine claudin-low mammary tumor cells is sufficient to induce a partial reversion to an epithelial morphology and to reduce cell migration. The miR-200b/200a/429 cluster but not the miR-200c/141 cluster was able to induce these alterations in cell shape and migration in the murine mammary tumor cell line, RJ423, while it was the miR-200c/141 cluster, and not the miR-200b/200a/429 cluster, that was capable of inducing these alterations in MDA-MB-231 cells. While cluster-specific effects in murine and human cell lines are possible, it is also possible that miR-200 expression levels are an important factor. In RJ423 cells, the miR-200b/200a/429 cluster was expressed at higher levels than the miR-200c/141 cluster while in the MDA-MB-231 cells the miR-200c/141 cluster was expressed at higher levels than the miR-200b/200a/429 cluster. Therefore, miR-200s may require expression levels beyond a certain threshold in order to have significant cellular effects. It also appears that if one miR-200 cluster is expressed at sufficiently high levels, it can positively influence the expression from the other miR-200 cluster. This was demonstrated in both RJ423ba429 where re-expression of the miR-200b/200a/429 cluster also induced a significant increase in miR-200c while in MDA-231c141 cells re-expression of the miR-200c/141 cluster induced a significant increase in miR-200b and miR-429 expression as determined by miRNA sequencing.

While the impact of miRNAs regulating the expression of mRNAs has been extensively investigated, the regulation of miRNAs by other miRNAs has largely been ignored. A small number of papers have shown that miRNAs can regulate other miRNAs [51–53], however, no paper describing a comprehensive examination of miRNAs regulated by the miR-200 family could be found. The miRNA sequencing results from our study demonstrate that simply re-expressing miR-200s induced alterations in the expression of a number of other miRNAs. Not only was the number of miRNAs regulated by miR-200 re-expression unexpected (> 100 miRNAs regulated in RJ423ba429 and MDA-231c141 cells), so was the small overlap (16–18%) of miRNAs regulated by the miR-200c/141 and miR-200b/200a/429 clusters in both the murine and human cancer cell lines. The small overlap in expressed miRNAs suggest that the miR-200c/141 and miR-200b/200a/429 clusters regulate the expression of different miRNAs.

Our observations that re-rexpression of miR-200s in claudin-low mammary tumor cells can inhibit proliferation and migration/invasion as well as altering cell shape is consistent with several published studies. These studies showed that re-expression of miR-200c in MDA-MB-231 cells significantly suppressed cell migration and promoted a more rounded cell morphology [54, 55], while treatment of MDA-231 cells with a miR-200c mimic was shown to significantly inhibit transwell invasion [56–59] or proliferation [57, 60]. Although our current study did not find that re-expression of the miR-200b/200a/429 cluster in MDA-MB-231 cells significantly impacted proliferation or invasion, other groups have shown that treating MDA-MB-231 cells with a miR-200a mimic [61], a miR-200b mimic [62–65], a miR-429 mimic [63], or overexpressing miR-200b [66], significantly reduced migration/invasion and treating MDA-MB-231 cells with a miR-200b or miR-429 mimic significantly reduced proliferation [63, 64]. It should be noted that two studies found miR-200 effects that contracted our findings. The first study stably expressed either the miR-200b/200a/429 or miR-200c/141 clusters in MDA-MB-231 cells and found that both clusters reduced cell growth (the miR-200c/141 cluster was more effective than the miR-200b/200a/429 cluster) and both clusters promoted migration and invasion [67]. The second study showed that re-expression of miR-200c significantly promoted invasion in a transwell assay [68]. Studies on murine claudin-low mammary tumor cells are far rarer and the only study outside our lab on murine claudin-low mammary tumor cells was performed in vivo and found that re-expression of the miR-200c/141 cluster in p53^null^ mammary tumors reduced tumor growth and metastasis to the lungs [69].

RNA sequencing revealed a similar pattern with RJ423ba429 and MDA-231c141 cells expressing more differentially expressed mRNAs than RJ423c141 or MDA-231ba429 cells, respectively. It also revealed that the miR-200c/141 and miR-200b/200a/429 clusters targeted distinct mRNAs despite both clusters sharing identical seed sequences. Cluster specific mRNA (and miRNA) expression profiles were observed in both murine and human mammary tumor cell lines reducing the possibility of this being an artifact. Another interesting observation from the RNA sequencing study was that no more that 12% of the differentially expressed mRNAs in any of the cell lines were predicted targets of the miR-200 family. This is not completely unexpected given the large number of miRNAs also regulated by the miR-200 family as described above. However, this data suggests that studies focusing only on predicted mRNA targets may miss a majority of mRNAs regulated by their miRNA of interest .

To reduce the complexity, differentially expressed mRNAs unique to RJ423ba429 and MDA-231c141 cells (the two lines showing changes in cell shape) were further examined. In both cell lines, the top ChEA pathway identified was SUZ12. SUZ12 is a component of the Polycomb repressive complex 2 (PRC2) that mono-, di- and trimethylates lysine 27 of histone H3 (H3K27) [43–50]. Trimethylation of H3K27 promotes chromosome compaction preventing transcription factors from accessing transcription start sites in promoters [70–72]. miR-200b and miR-200c have been reported to target SUZ12 [73–75], downregulating its expression and leading to hypomethylation of H3K27 and loss of H3K27 trimethylation. However, re-expression of the miR-200b/200a/429 cluster in RJ423 cells and re-expression of the miR-200c/141 cluster in MDA-MB-231 cells resulted in a significant increase in H3K27me3 levels. In normal mammary epithelial cells, higher levels of H3K27me3 are observed in mature luminal cells compared to mammary stem cells and H3K27me3 levels have been observed to increase during mammary epithelial differentiation during pregnancy [76]. Therefore, re-expression of the miR-200b/200a/429 cluster in RJ423 cells and re-expression of miR-200c/141 cluster in MDA-MB-231 cells may reduce the stem cell features of these cells and drive them to a more differentiated phenotype. Evaluation of gene specific H3K27 methylation patterns are required to confirm this and identify the genes affected.

## Conclusions

Understanding how miRNAs influence cellular function is already complex due to the large number of predicted targets and false positives in existing databases, however, our study demonstrates that miR-200s can regulate other microRNAs as well as epigenetic modulators thus adding additional complexity to this system. We have also shown that re-expression of miR-200s in claudin-low mammary tumor cells can revert claudin-low cells to a more epithelial phenotype provided the level of miR-200 re-expression is sufficiently high and this reversion is associated with alterations in SUZ12 regulated genes and reduced cell migration.

## Supplementary Information

**Additional file 1.** miRNA sequencing files for RJ423c141 vs. RJ423EV, RJ423ba429 vs. RJ423EV, MDA-231c141 vs. MDA-231ba429 and MDA-231ba429 vs. MDA-231EV cell lines.

**Additional file 2.** Venn diagrams showing unique and shared miRNAs in the mouse cell lines (A), the human cell lines (B), and a combination of the mouse and human cell lines (C).

## Data Availability

RNA sequencing has been uploaded to GEO under accession number GSE150107 and the miRNA sequencing files are presented as Additional file 1.

## Abbreviations

APC: Allophycocyanin
BrdU: 5-Bromo-2′-deoxyuridine
EMT: Epithelial to mesenchymal transition
ER: Estrogen receptor
FITC: Fluorescein isothiocyanate
H3K27: Lysine 27 of histone H3
H3K27me3: Trimethylated lysine 27 of histone H3
HER2: Human epidermal growth factor receptor 2
MDA-231ba429: MDA-MB-231 cells stably expressing the miR-200b/200a/429 cluster
MDA-231c141: MDA-MB-231 cells stably expressing the miR-200c/141 cluster
MDA-231EV: MDA-MB-231 cells stably expressing an empty vector
miRNA: microRNA
PCR2: Polycomb repressor complex 2
PR: Progesterone receptor
RJ423ba429: RJ423 cells stably expressing the miR-200b/200a/429 cluster
RJ423c141: RJ423 cells stably expressing the miR-200c/141 cluster
RJ423EV: RJ423 cells stably expressing an empty vector
TNBC: Triple negative breast cancer
