# Supplementary figures and images for: Re-expression of miR-200s in claudin‐low mammary tumor cells alters cell shape and reduces proliferation and invasion potentially through modulating other miRNAs and SUZ12 regulated genes
Source: Cancer Cell Int. 2021 Feb 4;21:89. doi: 10.1186/s12935-021-01784-4 (PMC7863273; doi:10.1186/s12935-021-01784-4)

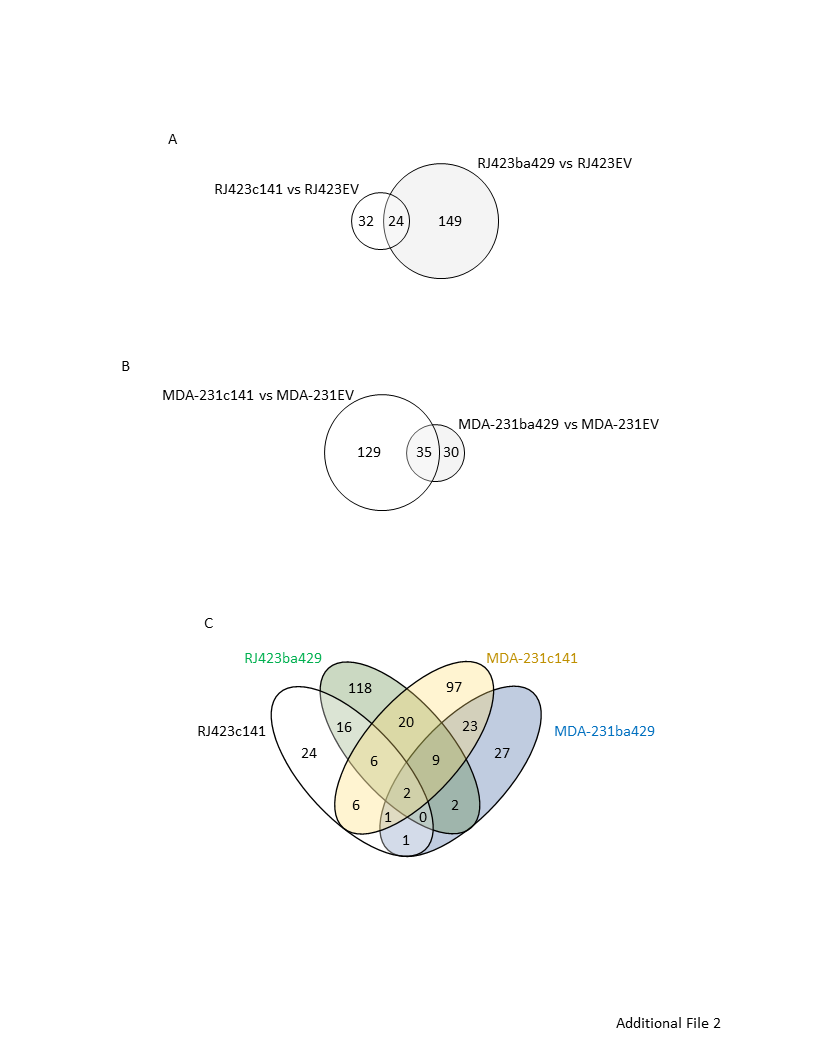

Supplement: Supplementary file 2 — Additional file 2. Venn diagrams showing unique and shared miRNAs in the mouse cell lines (A), the human cell lines (B), and a combination of the mouse and human cell lines (C). [file 12935_2021_1784_MOESM2_ESM.tif]
